# Occult Hepatitis B Virus Infection in Anti-HBs-Positive Infants Born to HBsAg-Positive Mothers in China

**DOI:** 10.1371/journal.pone.0070768

**Published:** 2013-08-12

**Authors:** Haixia Su, Yuhai Zhang, Dezhong Xu, Bo Wang, Lei Zhang, Duan Li, Dan Xiao, Fan Li, Jingxia Zhang, Yongping Yan

**Affiliations:** 1 Department of Epidemiology, Fourth Military Medical University, Xi'an, Shaanxi, China; 2 Department of Health Statistics, and the Ministry of Education Key Lab of Hazard Assessment and Control in Special Operational Environment, Fourth Military Medical University, Xi'an, Shaanxi, China; University of North Carolina School of Medicine, United States of America

## Abstract

**Objective:**

To investigate the prevalence of occult HBV infection (OBI) among children and to characterize virology of occult HBV, we conducted an epidemiological survey.

**Methods:**

186 HB-vaccinated infants born to HBsAg-positive mothers were included in the study. Serological tests for HBV markers were performed using commercial ELISA kits. Real-time quantitative PCR and nested PCR were used to detect HBV DNA. PCR products of the C and pre-S/S regions were sequenced and analyzed.

**Results:**

1.61% (3/186) infants were HBsAg positive, and 4.92% (9/183) infants were considered as occult infection. The viral load of mothers was associated with occult infection (*P* = 0.020). Incomplete three-dose injections of HB vaccine was associated with HBV infection (*P* = 0.022). Six OBI infants were positive for anti-HBs, but their titers were not greater than 100 mIU/mL. Seven isolated HBV pre-S/S sequences were obtained from nine OBI infants. Three of the sequences were genotype C, and four of the sequences were genotype C/D. Escape mutation S143L was found in the four sequences of genotype C/D. All seven sequences lacked G145R and other escape mutation in S region.

**Conclusions:**

Occult HBV infection was detected in anti-HBs positive infants born to HBsAg-positive mothers in China. Occult infection was associated with absent anti-HBs or with low anti-HBs level, high maternal viral loads and escape mutations in the S gene.

## Introduction

Hepatitis B virus (HBV) infection is a major global health problem, causing chronic hepatitis, cirrhosis, hepatocellular carcinoma (HCC) and other chronic liver diseases [1], [2]. China is a highly endemic area for HBV infection [3], [4], [5]. The hepatitis B surface antigen (HBsAg) carrier rate was 7.18% for the overall population by a seroepidemiological survey on HBV infection in 2006 [6]. In infants 1 to 4 years of age, the HBsAg carrier rate decreased to 0.96% in 2006 after the implementation of universal HB vaccination from 1992 [6]. Although the hepatitis B vaccine is safe, cost-efficient and effective at preventing infection and controlling the spread of hepatitis B, HBV breakthrough infection may occur in anti-HBs-positive or HBsAg-negative vaccinees [7], [8], [9], [10].

Occult HBV infection (OBI) is defined as the persistence of viral genomes in the liver tissue or the serum of individuals who are HBsAg-negative [11],[12]. The occult status of infection may be due to host immunosuppression, co-infection with hepatitis C virus (HCV), the window period after acute infection, genetic mutations in the S gene, or other host factors [11],13],[14]. The clinical consequence and transmission risk of OBI in populations remains unclear. OBI may persist in individuals for years without obviously symptoms of overt HBV infection [15] or may develop in to hepatitis, cirrhosis and HCC [14],[16].

Occult HBV may be transmitted by blood transfusion or organ transplantation [17]. Some studies also suggested that occult HBV may be transmitted between relatives or transmitted to children from occult infected or HBsAg-positive mothers [18],[19]. Given these potential transmission routes, occult infection in children cannot be neglected. There have been few previous studies on OBI in children. Some of these detected HBV DNA in children with anti-HBs or anti-HBc and identified a few immune escape mutations in the ‘a’ determinant of HBsAg. In Taiwan, a pilot study indicated that the prevalence of occult HBV infection was 10.9% in HBV-vaccinated children from the pediatrics department of a hospital [9]. In Iran, a small sample study estimated that the prevalence of OBI was 28% in immunized children born to HBsAg-positive mothers [8]. In China, there is a high incidence of chronic HBV infection, and approximately 7% of pregnant women are HBsAg-positive. However, little is known about occult infection among children born to HBsAg-positive mothers, despite the fact that the HBsAg carrier rate of children was decreased by neonatal HB vaccination. Here, we conducted an epidemiological survey among HB-vaccinated infants in a highly endemic area to investigate the prevalence of occult infection among children and characterize the serology and virology of occult HBV.

## Materials and Methods

### Study Population

Wuwei City is one of the most under-developed areas in northwestern China. From 2007 to 2011, the average reported incidence rate of hepatitis B was 634.56/100,000 people, significantly higher than the national average (88.82/100,000 people) [20]. The HBsAg-positive rate among pregnant women in Wuwei City has been reported to be approximately 7.11% [3]. Their newborns of HBsAg-positive pregnant women were immunized with 5 µg recombinant yeast-derived hepatitis B vaccine (Shenzhen Kangtai Biological Products Company, Shenzhen, China) following a 0-, 1-, and 6-month vaccination schedule. HBV immunoglobulin (HBIG, 100 IU) also were given within 24 h of birth. From 2009–2010, there were 326 HBsAg-positive pregnant women from 3 main hospitals in Wuwei City. Among them, 73 HBsAg-positive mothers could not be contacted because of wrong telephone number or absent telephone, 55 HBsAg-positive mothers rejected to participate in the study, and 3 mothers were excluded for HBsAg-negative after re-tested by ELISA. Then the remaining 195 HBsAg-positive mothers and their 202 infants were recruited. Our study was approved by the ethics committee of the Fourth Military Medical University. All parents signed an informed consent form and provided the child's vaccination history based on a vaccination booklet. Sixteen infants were excluded for lack of hepatitis B vaccination or absent vaccination information, yielding a final study cohort of 181 HBsAg-positive mothers and their 186 infants. All infants had finished the first two or three doses of the hepatitis B vaccination schedule. The age of infant was calculated at the time of infant enrolment in the study. Venous blood specimens from mothers and their infants were collected. Sera were separated and stored at −80°C for future testing.

### Serological Assays

HBsAg-positive mothers were tested for HBsAg and HBeAg, and their infants were tested for HBsAg, anti-HBs and anti-HBc, using commercial ELISA kits (Kehua Biotechnology, Shanghai, China) according to the manufacturer's instructions. The sensitivity of the assays for serological markers was 0.05 ng/mL. HBsAg-negative and HBV DNA positive serum specimens were re-tested for HBsAg and anti-HBs using reagents from Abbott Laboratories (Abbott Park, IL, USA). Titers of anti-HBs <10 mIU/mL were considered negative. The sera of mothers were also screened for antibodies of hepatitis C virus (anti-HCV) using commercial ELISA kits (Kehua Biotechnology, Shanghai, China) according to the manufacturer's instructions. The HIV infection of HBsAg-positive mothers was not assessed.

### HBV DNA Extraction

HBV DNA extraction was performed using TIANamp Viral DNA/RNA kits (TIANGEN Biotechnology, Bei-Jing, China) according to the manufacturer's instructions. The viral lysate was prepared by adding 20 µL of proteinase k (20 mg/mL) to a 200 µL aliquot serum sample, followed by addition of 200 µL carrier RNA buffer; this mixture was incubated at 56°C for 15 minutes. After brief centrifugation, 250 µL of 100% ethanol was added to each well and incubated for five minutes at room temperature. Then, the lysate was transferred to a column and briefly centrifuged. The column was washed two times by 500 µL of wash buffer and then once by 500 µL of 100% ethanol. Finally, 50 µL of RNase-free water was added to each column, and the eluted DNA was stored at −80°C.

### Quantification of HBV

Real-time fluorescence-based quantitative PCR was used to measure HBV DNA levels in serum samples. Sample handling procedures were in strict accordance with the reagent kit instructions (Daangene Biotechnology, Guangzhou, China).The detection range for HBV DNA was from 1×10^2^ to 10^8^ IU/mL. According to the instructions, an HBV DNA level above 1.0×10^2^ IU/mL was considered a positive result.

### Nested PCR Amplification

Nested PCR was performed using specific primers (Table 1) targeting the S, C and P regions of the HBV genome. All primers were synthesized by Sunny Biotechnology Co., Ltd (Shanghai, China). The sensitivity of the PCR assay was determined by serial dilutions of serum samples containing known concentrations of the HBV genome: 1×10^4^, 1×10^3^, 5×10^2^, 1×10^2^ IU/mL. The detection limit for the nested PCR assay was approximately 1×10^2^ IU/mL. Serum samples that contained 2 random fragments from the S, C and P regions were considered positive. Amplification was performed for 30 cycles as follows: denaturation at 94°C for 30 s, annealing at 55°C for 50 s and elongation at 72°C for 1 min; after these cycles, an extra incubation at 72°C for 5 min was performed to ensure full extension of the products. Another PCR was then performed using a Takara LA PCR kit Ver. 2.1 (TAKARA Biotechnology, Da-Lian, China) to obtain a 1.2 kb fragment of the HBV genome including the pre-S1, pre-S2 and S regions for sequence analysis. The PCR conditions were as follows: 5 min at 94°C followed by 35 amplification cycles of 45 s at 94°C, 1 min at 60°C and 2 min at 72°C. Precautions were taken to avoid cross-contamination during sample collection, DNA extraction, PCR mix preparation, and gel electrophoresis. Negative controls were included in each assay.

**Table 1:** Nested PCR primers and 1.2 kb PCR primers used in this study.

Primer	Location	Sequences	Application
HBV-S1	57-74	CTGCTGGTGGCTCCAGTT	S gene External [36]
HBV-S2	756-739	CAATACCACATCATCCAT	
HBV-S3	187-204	CACTGCAGCCTGCTCGTGTTACAGGC	S gene Internal [36]
HBV-S4	687-669	CGGTCGACGGCACTAGTAAACTGAGC	
HBV-C1	1818-1841	AACTTTTTCACCTCTGCCTAATCA	Pre-C/C gene External [35]
HBV-C2	2452-2429	CTA ACATTGAGATTCCCGAGATTG	
HBV-C3	1838-1855	CCGGATCCTCTCATGTTCATGT	Pre-C/C gene Internal [35]
HBV-C4	2412-2393	CGAAGCTTGAGATCTTCGTCT	
HBV-P1	2413-2433	CGTCGCAGAAGATCTCAATC	P gene External
HBV-P2	174-154	CCTGATGTGATGTTCTCCATG	
HBV-P3	2457-2473	CCTTGGACTCATAAGGT	P gene Internal
HBV-P4	2984-2965-	TTGAAGTCCCAATCTGGATT	
HBV-S5	2826–2844	CGGGATCCCATATTCTTGGGAACAAG	1.2-kb pre-S/S gene [36]
HBV-S6	839–821	CACTGCAGGGTTTAAATGTATACCCA	

### Criteria for Occult HBV Infection

Only subjects who tested negative for HBsAg but positive for HBV DNA by nested PCR or real-time quantitative PCR were considered positive for occult HBV infection.

### Nucleotide and amino acid analysis

The PCR products in the core region and in the pre-S/S region were directly sequenced by Sunny Biotechnology Co., Ltd (Shanghai, China) using an automated DNA sequencer (ABI 3730). Alignment and multiple comparisons of HBV sequences from OBI infants with Genbank reference sequences (genotypes A, B, C and D) were performed by the ClustalW program integrated in MegAlign software. Phylogenetic analysis was performed by MegAlign software in DNASTAR software package (DNASTAR Inc., Madison, WI, USA). Accession numbers of the 4 reference sequences were as follows: X02763, D00329, M12906, X02496. Genotyping and subgenotyping of HBV was performed by phylogenetic analysis of the S gene with sequences from the database. S gene sequences were also used to determine serosubtypes.

### Statistical Analysis

Age value is expressed as the mean±standard deviation (SD). The frequency was compared between groups using the chi-squared test, *t*-test or Fisher's exact test. Statistical tests were 2-tailed, and a *P* value<0.05 was considered statistically significant. An odds ratio with a 95% confidence interval was denoted for each analysis. All procedures were performed using SPSS for Windows version 16.0 (SPSS Inc., Chicago, IL, USA).

## Results

### Occult HBV Infection Prevalence

**Figure 1:**
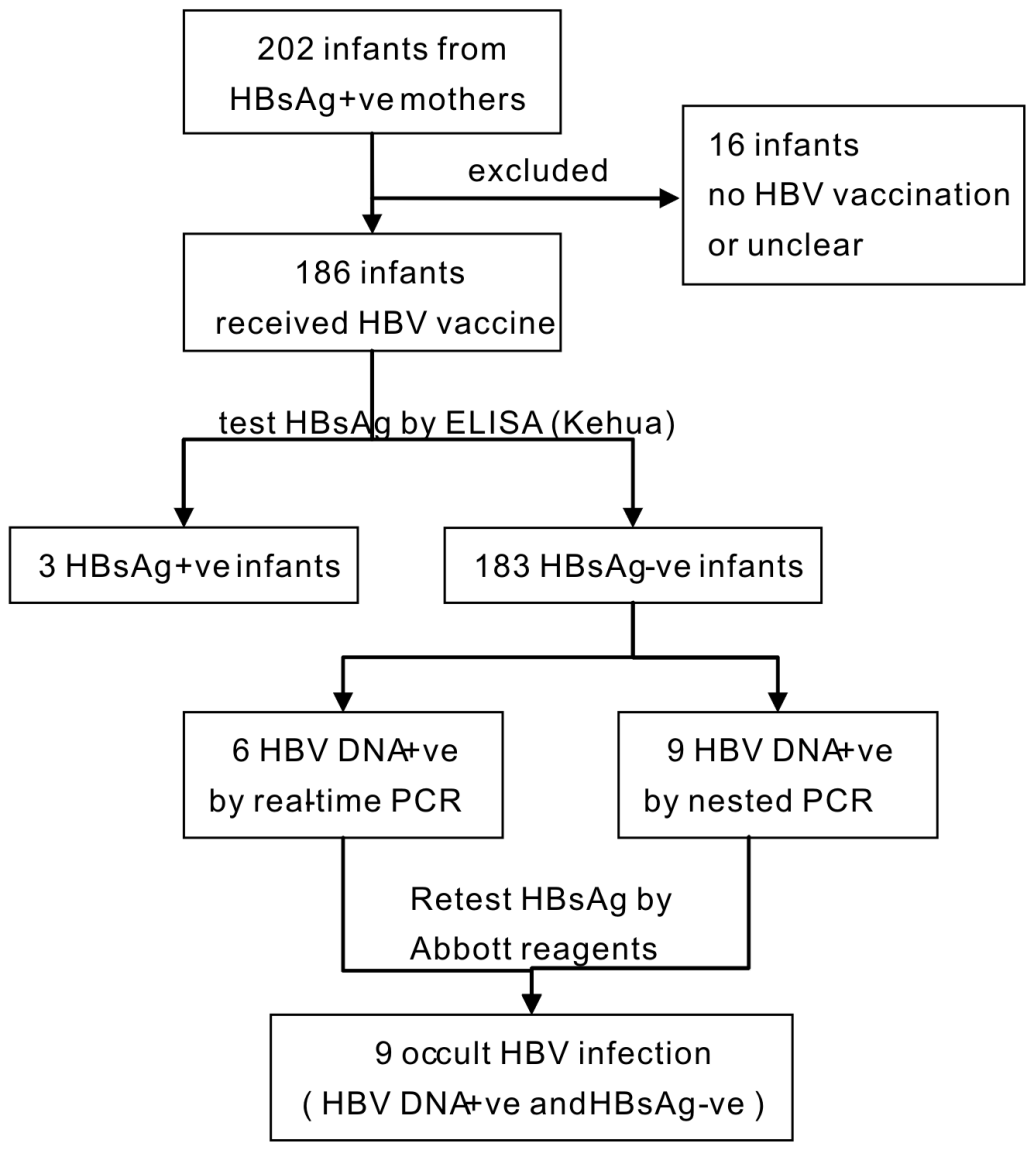
Diagram showing the diagnostic workflow of tests for identifying occult HBV infection in infants (+ve, positive; −ve, negative).

We studied 186 infants who received HB vaccination and their 181 HBsAg-positive mothers. The age of the infants ranged from one to 51 months, with a mean age of 16.05±13.06 months, and there were 99 (53.22%) males and 87 (46.77%) females. The 186 infants had finished two-dose or three-dose injections of HB vaccine according to their vaccination schedule, and 128 (68.81%) had received HBIG injections (100 IU) after birth. HBsAg was detected by ELISA using Kehua assay, and 3 infants tested positive. Then, HBV DNA was detected by real-time PCR and nested PCR in the other 183 infants who were negative for HBsAg (Fig. 1). Six infants had detectable HBV DNA, and their viral loads ranged from 10^3^ to 10^7^ IU/mL (Table 2). We also performed nested PCR after real-time PCR and obtained gene fragments of HBV for sequencing. Five infant sera were positive for both the S and C genes fragments, 2 were positive for both the P and C genes fragments, and 2 were positive for the S, P and C genes fragments (Table 2). Altogether, 6 infants tested positive for HBV DNA by real-time PCR and nested PCR. Another 3 infants had HBV DNA that were only detectable by nested PCR. The 9 infants identified as HBV DNA positive were re-tested for HBsAg by Abbott reagents; none were positive. We obtained 9 infants with OBI who tested positive for HBV DNA but negative for HBsAg in the serum. The prevalence of OBI in infants received neonatal HB vaccination from HBsAg-positive mothers was 4.92% (9/183), with a 95% CI of 1.79% to 8.05%. Other serological markers were also assayed in the subjects. Among the 186 infants, 77.96% (145/186) had anti-HBs and 33.87% (63/186) had anti-HBc.

**Table 2:** HBV DNA levels in 9 infants diagnosed with occult HBV infection.

Code	HBV DNA (IU/mL)	Nested PCR		
		S gene	Pre-C/C gene	P gene
2001	29300000	+	+	+
2004	7720	+	+	+
2005	42700	+	+	−
2008	9630	+	+	−
2058	<100	+	+	−
1012	1490	+	+	−
2015	9600	+	+	−
2067	<100	−	+	+
3039	<100	−	+	+

### Factors Associated with Occult HBV Infection in Infants

The demographics, serological markers and epidemiological data for the 9 occult infection infants are listed in Table 3. These 9 infants included 2 females and 7 males, and their mean age was 11.00±13.06 months. There were no significant differences in the age and gender ratio between OBI-positive and OBI-negative infants (Table 4, *P*>0.05). Although all infants had been immunized with two-dose and three-dose HB vaccine according to vaccination schedule, HBIG was not administered to every infant. Two of 9 (22.22%) OBI-positive infants and 54 of 174 (31.03%) OBI-negative infants did not receive HBIG after birth. HBIG usage and HBV vaccine injection times were not associated with occult infection. In HBV infected infants including 3 HBsAg-positive and 9 OBI infants, 66.66% (8/12) received two doses vaccine, which was obvious higher than non-infection infants (31.03%, 54/174). It showed that incomplete three-dose injections of HB vaccine was associated with HBV infection (*P* = 0.022).

**Table 3:** Demographics, serological markers and epidemiological data for the 9 OBI infants.

Code	Gender	Age (months)	HB vaccine injection times	HBIG	Anti-HBs (mIU/mL)	Anti-HBc	Maternal HBeAg	Maternal HBV DNA (IU/mL)
2001	Male	1.55	2 dose	No	-	**+**	+	4.0E8
2004	Male	38.40	3 dose	Yes	-	**−**	−	145000.0
2005	Male	2.05	2 dose	Yes	32	**−**	−	<100
2008	Male	1.06	2 dose	Yes	13	**−**	−	7920.0
2058	Male	2.01	2 dose	Yes	28	**−**	−	<100
1012	female	7.65	3 dose	Yes	61	**−**	−	2060.0
2015	Male	6.14	2 dose	Yes	97	**−**	−	9680.0
2067	female	14.39	3 dose	Yes	59	**−**	+	7.23E7
3039	Male	25.79	3 dose	No	**-**	**−**	−	<100

**Table 4:** Univariate analysis of factors associated with occult HBV infection among HB-vaccinated infants.

Factors	OBI-positive infants (n = 9)	OBI-negative infants (n = 174)	*p*	OR (95% CI)
Age (months)	11.00±13.06	16.44±13.04	0.228	
Gender (male/female)	7/2	90/84	0.176	3.27 (0.66,16.17)
HB vaccine injection times^1^ (2 doses/3 doses)	5/4	54/120	0.150	2.78 (0.72,10.75)
HBIG	7	120	0.724	1.58 (0.32,7.83)
Anti-HBs positivity	6	138	0.404	0.52 (0.12,2.19)
Anti-HBc positivity	1	62	0.167	0.23 (0.03,1.84)
Maternal HBeAg positivity	2	29	0.650	1.43 (0.28,7.23)
Maternal HBV DNA load >100 IU/mL	6	48	**0.020**	**5.25** (1.26,21.83)

1 stand for finished two-dose or three-dose injection according to vaccination schedule.

The viral load of mothers was associated with occult infection (Table 4, *P* = 0.020); the percentage of maternal viral loads >100 IU/mL (66.66%) in OBI-positive infants was significantly higher than that of OBI-negative infants (27.59%, *P* = 0.020). Other serologic markers of mothers or infants were not significantly different between OBI-positive and -negative infant groups. Of note, none of the pregnant mothers had detectable HCV antibodies. Three of the pregnant mothers received liver-protective therapy, whose infants were not infected by HBV, but none received specific antiviral treatment in pregnancy.

Only one OBI infant (2001) tested positive for anti-HBc, and his HBV DNA load was 2.93×10^7^ IU/mL. His mother was positive for HBeAg and also had high viral load, 4.00×10^8^ IU/mL. The level of anti-HBs in OBI infants was low: 6 infants tested positive for anti-HBs, but their titers were less than 100 mIU/mL; the other 3 OBI infants were negative for anti-HBs (Table 3).

### Mutations in the Viral Genomes Isolated from OBI Infants

**Figure 2:**
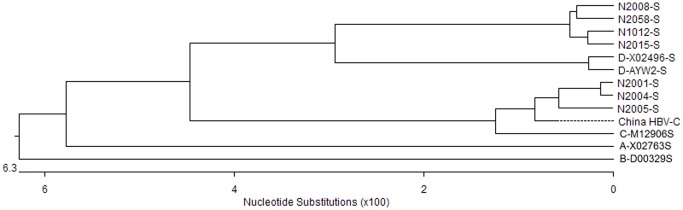
Phylogenetic tree constructed on the pre-S1, and pre-S2 and S gene of 6 reference nucleotide sequences and 7 nucleotide sequences isolated from 7 occult HBV infection infants. Reference sequences of HBV genotypes are denoted according their GenBank accession number. China HBV-C is the HBV reference sequence of Chinese with C genotype.

**Figure 3:**
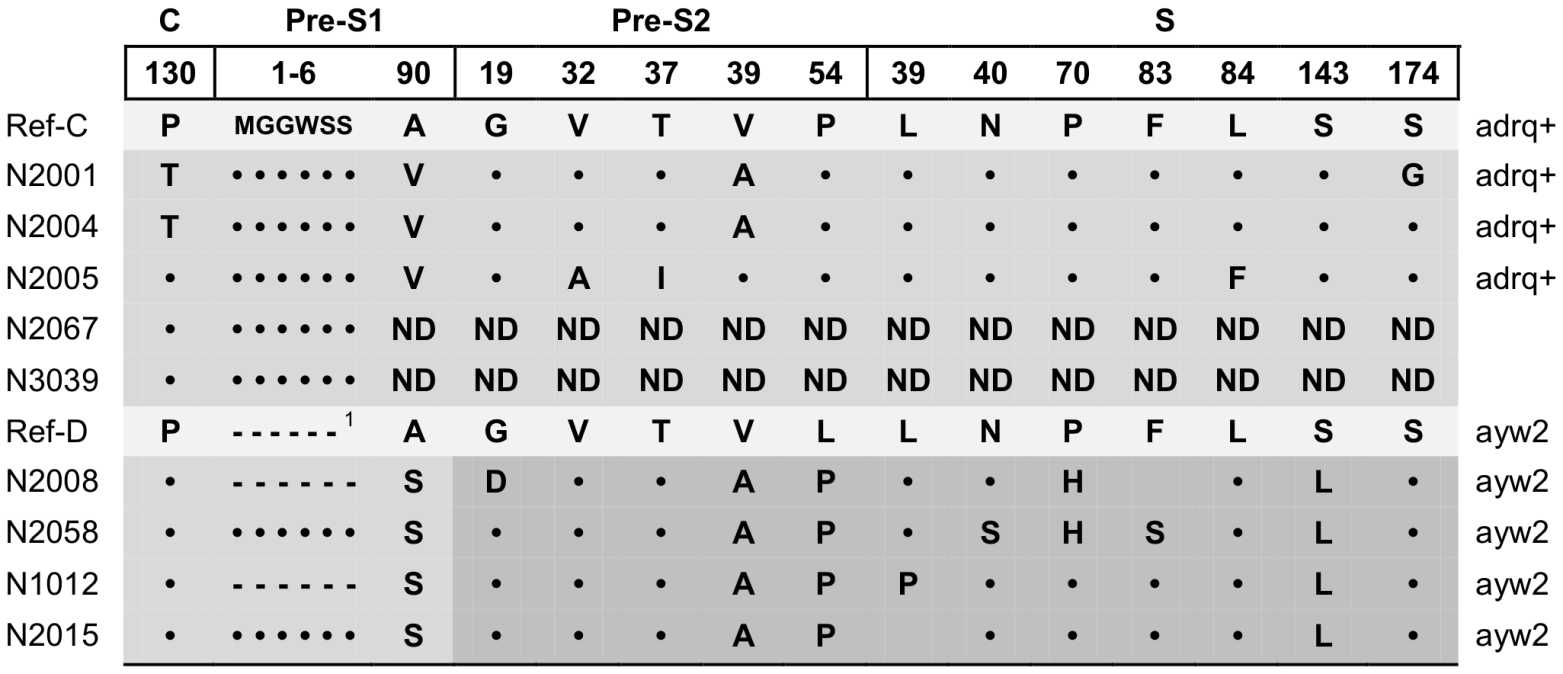
Mutations in the 9 OBI isolates in the C, pre-S1, pre-S2 and S regions. The first three sequences were compared with the reference sequence of genotype C. The other four sequences were compared with the reference sequence of genotype C in the pre-C/C and pre-S1 regions and reference sequence of genotype D in the pre-S2 and S regions. •: Amino acid identical with reference sequence. -: Amino acid deleted. ^1^: 11 amino acid deletion in the pre-S1 region of the genotype D reference. ND: sequence were not obtained.

The HBV core regions and the pre-S/S regions of 7 infant samples were successfully sequenced. According to the hylogenetic tree (Figure 2) and alignment results, three infants carried genotype C and serotype adrq+. Another four infants carried the recombinant genotype C/D and serotype ayw2. The four sequences of pre-C/C and pre-S1 regions closed with genotype C, while the sequences of pre-S2 and S regions closed with genotype D. The other 2 infants (2067 and 3039) only obtained C and P regions sequences. The mutations in 9 OBI infant isolates were analyzed by comparison with reference sequences of genotypes C (M12906) and D (X02496). Mutations found in more than two infants were shown in Figure 3, and those found in only one infant were excluded. P130T in the C region and A90V/S in the pre-S1 regions were observed in 2 and 6 isolates, respectively. A 6 amino acid deletion in the pre-S1 regions was also found in 2 isolates of genotype C/D. In the pre-S2 region, V39A and L54P were observed in 6 and 4 isolates, respectively. In the S region, P70H and S143L were found in 2 and 4 isolates, respectively. Mutation S143L was found in four sequences of genotype C/D in the “a” determinant, which is also related to vaccine escape. All seven sequences lacked G145R and other escape mutation in S region.

## Discussion

Occult HBV infection was first reported in the late 1970s and has received more attention in the past ten years. Researchers have been mostly interested in OBI in transfusion and organ transplantation; therefore, their study subjects were typically donors or patients with chronic hepatitis or other chronic liver diseases. The studies on OBI in general populations or children were a few, and the reported prevalence of OBI varied between different detection methods and different endemic regions. China is a highly endemic region for chronic hepatitis. The OBI in adults has been detected in approximately 1% to 3% of vaccinees [7],[10]. In children, occult infection likely exists but its prevalence rate is unclear. HBV infection is easier to occur in infancy and childhood. Once infants are infected by HBV, 25–30% will become chronic carriers for life [21] [22], and they have a higher risk of developing liver cirrhosis and liver cancer, compared to patients infected in adulthood. Therefore, we surveyed 186 high-risk infants born to HBsAg-positive mothers, detected their serum HBV DNA levels and analyzed HBV genome sequences obtained from infants with occult infection. Although all infants had received HB vaccination after birth, 9 infants tested positive for OBI. The prevalence of OBI in our study was 4.92% (9/183). The total HBV infection rate, including HBsAg-positive infection and occult infection, was 6.6% (12/186) in vaccinated, high-risk infants over 4 years old. Based on our data, we thought HB vaccination in neonates is an efficient prevention strategy and should be implemented continuously.

However, the mechanism of OBI in vaccinated children is unclear. In general, OBI may represent the window period of acute infection, persistence of low level replication after recovery, or the occurrence of an escape mutant undetected by current HBsAg assays [23]. Many factors may be related to occult infection in vaccinated children, such as nonresponse or hyporesponse to HB vaccination, declining titers of protective antibody, escape mutations in the S gene, or high maternal viral loads [11].

In our study, we found that maternal viral loads were associated with occult infection. Other factors, including gender, age, vaccine dose, anti-HBs, anti-HBc and HBeAg seroconversion of the mother, were not significantly different between the OBI-positive and -negative groups. The maternal viral load was consistently a major risk factor for HBV infection, regardless of HBsAg-positive infection or occult infection. Mothers with higher viral loads had greater risk for mother-to-infant transmission. Therefore, in highly endemic areas, neonates from HBsAg-positive mother should immediately receive the HB vaccine and HBIG within 24 hours after birth, according to vaccination schedule against HBV infection. HBsAg-positive pregnant women with high viral loads (>10^8^ IU/mL) may consider antiviral treatment in the third trimester to prevent perinatal HBV transmission. Co-infection with HCV was not found in HBsAg-positive mothers. Because the rate of HIV infection is very low in Wuwei, HIV co-infection in mothers was not detected. The possible influence of co-infection on HBV occult infection was not analyzed in this study.

The positivity of anti-HBs or titers of anti-HBs greater than 10 mIU/mL are usually considered to produce protective immunity against hepatitis B. Despite this protection, HBV infection also could be detected in anti-HBs-positive individuals [12],[24]. In this study, 3 OBI infants were negative for anti-HBs; 6 infants were positive for anti-HBs; their titers were lower than 100 mIU/mL. These data showed that the anti-HBs levels were low in OBI infants. Several studies have also suggested that the neutralizing capacity of low-level anti-HBs is limited [10],[25],[26]. The low anti-HBs titers or absent anti-HBs may be related to serovaccination failures, incomplete 3-dose vaccines or intrauterine infection [26]. In our subjects, all infants received 5 µg per dose of recombinant yeast HB vaccine. A large population study in China showed that 10 µg HB vaccine had a higher prophylaxis effective rate than 5 µg [27], and the 10 µg dose was suitable for newborns [28], and it was also recommended by chronic hepatitis B prevention and treatment guidelines (2010, China). To induce higher concentrations of anti-HBs and more robust protective immunity, we suggest an increase in vaccine dosage to 10 µg for neonates born to HBsAg-positive mothers. Moreover, these high-risk infants should be timely tested for HBsAg and anti-HBs titers after completion of 3 doses of HB vaccine. If anti-HBs levels are low or negative, infants should then be given an HB vaccine booster to decrease the risk of HBV infection.

Occult HBV infection of infants possibly comes from vertical transmission or mother-to-infants transmission. Loss of HBsAg or HBV DNA may occur in a proportion of vertically exposed infants within the first year of life. In our study, 6 OBI infants were less than one-year old. Because the sera of neonates at birth time and sera after 1 year old were not obtained, it is really difficult to distinguish vertical infection from occult infection. In the future study, we will establish a neonate cohort, continuously collect sera of infants from birth to 2 years old, and follow-up occult infection course and outcome.

Mutations in ‘a’ immunodeterminant of the surface protein may decrease immune recognition of the virus and cause diagnostic failure [12]. G145R is the most common escape mutation in the surface protein [29]. In Iran, 10 of 14 OBI isolates from children (71%) contained the G145R mutation [8]. No G145R mutations were found in OBI isolates from children in the present study or a previous study in Taiwan [30]. The differences in these results may be associated with the geographical distribution or genotypes [31]. Mainland China, Hong Kong and Taiwan are highly endemic regions, and the major genotypes in these areas are C and B. Among OBI adults and overt infection patients in these regions, few had the common HBsAg mutant G145R [10],[32]. Recent studies suggested that other mutations in the surface protein were associated with OBI. Svicher et al. [33] identified the OBI-associated mutations Y100S, T116N, R122P, S143L and S167L from a large-sample, blood-donor study. These mutations in genotype D or other genotypes strongly affected HBsAg detection by altering HBV-antigenicity or viral-particle maturation [31]. HBV isolates with ‘a’ determinant mutations were not efficient neutralized by anti-HBs antibody induced by HB vaccine [29]. In this study, 4 OBI infants with C/D genotype had S143L mutation, and their HBsAg-negative occult state may be attributed to the escape mutation. Other common escape mutations, including G145R, D144A, P142S, Q129H, I/T126N/A and M133L [29], were not found in our OBI isolates. Other escape mutants associated with antiviral resistance [34] were not found because their HBsAg-positive mothers did not receive antiviral treatment in pregnancy. In the beginning of the pre-S1 coding region, 18 nucleotide (6 amino acids) deletions were found in 2 isolates with the C/D genotype. These deletions may impair viral packaging, cause structural alterations in genomic regulatory regions and could result in a significant reduction in HBsAg expression [35]. In the other 3 OBI isolates with genotype C, these escape mutations and deletions were not detected. Their occult infection status may be attributed to strong suppression of HBV replication and gene expression [11],[12].

In conclusion, the prevalence of OBI in HB-vaccinated infants from HBsAg-positive mothers was 4.92% in China. Occult infection was associated with absent anti-HBs or with low anti-HBs level, high maternal viral loads and escape mutations in the S gene. These results suggest that the HBV neonatal immunization strategy in our country should be strengthened and supplemented for newborns from HBsAg-positive mothers by increasing vaccine dosage, ameliorating vaccine for escape mutations, and timely testing of HBsAg and anti-HBs titers after immunization.

However, it was a pilot study with small sample size, 9 occult infection infants were not adequate for risk factors analysis and multiple comparison of HBV sequences. In the further study, we will try to obtain more occult infection infants, compare their viral sequences with that of HBsAg-positive infants, and explore the sensitivity of occult infection isolates to anti-HBs in order to elucidate occult infection mechanism.
